# Digital innovations and health information systems: Lessons learned from implementing ALMANACH in Nigeria

**DOI:** 10.1371/journal.pdig.0001362

**Published:** 2026-06-22

**Authors:** Andrea Bernasconi, Daniel Ishaya, Ibrahim Sahabo, Muazu Muazu, Marianne van der Sande

**Affiliations:** 1 Institute of Tropical Medicine Antwerp, Antwerpen, Belgium; 2 Julius Global Health, University Medical Center Utrecht, Utrecht, The Netherlands; 3 Salus Mundi, Moutier, Switzerland; 4 Malaria Consortium, Maitama, Abuja- Federal Capital Territory, Nigeria; 5 Adamawa State Primary Health Care Development Agency, Jimeta, Nigeria; University of British Columbia, CANADA

## Abstract

In low- and middle-income settings, routine health information systems (RHIS) and digital health projects often coexist, but their epidemiological outputs are rarely compared—even though integrating digital tools could strengthen RHIS by reducing reporting challenges. We conducted a retrospective, facility-level analysis of quarterly data (2017–2021) from Adamawa State, Nigeria, comparing ALMANACH, a clinical decision support system used in primary health care, with the state RHIS for malaria, pneumonia, gastrointestinal disorders, and measles in children under five years of age. The primary outcome was the facility-aggregated quarterly absolute relative difference (ARD) between the two reporting systems; temporal trends and facility-level heterogeneity were also assessed. Paired non-parametric tests, effect sizes with 95% confidence intervals (95% CI), and linear mixed-effects models accounted for clustering and repeated measurements. Across the study period, ALMANACH reported fewer cases than RHIS for malaria (116,018 vs 233,548; ARD 80.6%, 95% CI: 64.4–89.6, p < 0.01), gastrointestinal disorders (43,003 vs 62,412; ARD 32.6%, 95% CI: 17.1–47.4, p < 0.01), and pneumonia (20,980 vs 29,416; ARD 34.0%, 95% CI: 32.3–48.0, p < 0.05), but more measles cases (4,355 vs 2,508; ARD 86.2%, 95% CI: 45.6–162.8, p < 0.01). Mixed-effects models showed that RHIS recorded, per facility-quarter, on average 40.2 (95% CI: 31.1–49.3) more malaria, 6.6 (95% CI: 4.3–8.0) more gastrointestinal disorders, and 2.9 (95% CI: 1.2–4.6) more pneumonia cases than ALMANACH (all p < 0.01), while ALMANACH reported 0.6 (95% CI: 0.07–1.2) more measles cases (p < 0.05). Divergence widened as ALMANACH scaled up. Without a gold standard, these results quantify discrepancies without implying superiority and highlight the need for data integration, harmonized case definitions, and stronger data-use practices. They underscore the importance of sustained stakeholder engagement and user involvement for effective digital health scale-up in resource-limited settings.

## Author summary

Reliable health data are essential for improving healthcare, yet many Low- and Middle-income Countries struggle to collect accurate information. Digital tools are often introduced to strengthen quality of care, but it is unclear how the data from these systems compare with those official from existing government records. In this study, we compared five years of childhood disease reports from a digital platform used in primary health in Adamawa State, Nigeria, with the state’s routine health information system. We found large and consistent differences in the number of cases reported for malaria, pneumonia, gastrointestinal infections, and measles, and these differences grew as the digital intervention scaled up. In the absence of a gold standard, our results cannot identify which system is more accurate. Rather, they point to the need for closer coordination, harmonised definitions, and improved practices for data collection and use. Strengthening these areas, and actively involving health workers and local decision makers, is key to ensuring that digital health projects improve care and guide policy in resource-limited settings.

## Introduction

Routine Health Information Systems (RHIS) are critical tools to strengthen health systems [1,2] and are considered one of its six core functions [3]. Defined as “*any system of data collection, distribution, and use that provides information at regular intervals*” [1], RHIS capture key information on health status, service utilization, and health resources within a population. These data are essential for informing decision-making, enabling efficient resource allocation, day-to-day management, strategic planning, and policy development [4–8].

Despite their critical role, RHIS in many LMICs face persistent fragmentation, weak data management, and poor interoperability, undermining data quality, timeliness, and representativeness [9–11]. Bottlenecks can occur at any stage of data collection and use, while deficiencies in technical capacity, infrastructure, and system integration further compromise data reliability [12,13]. On the behavioural side, low utilization of RHIS data and a lack of motivation among frontline staff hinder the translation of information into actionable insights [14]. Chronic underinvestment, weak leadership, and a poor culture of data use perpetuate suboptimal governance and decision-making [6,15].

These persistent challenges force health managers to rely on intermittent cross-sectional population-based surveys to monitor population health, risk factors, and service coverage [16]. Even critical service such as the Expanded Program on Immunization have shown marked discrepancies between RHIS-reported coverage and estimates from demographic and health surveys [17]. Furthermore, underperforming RHIS obscure progress on key indicators, limiting the ability to demonstrate impact and secure funding [18–21].

The steadily and progressive introduction of Information and Communication Technology (ICT) may partially bridge these gaps by enabling automatic and near real-time data capture [22]. In 2016, a partnership between the Swiss Tropical and Public Health Institute (Swiss TPH), the International Committee of the Red Cross (ICRC), and the Adamawa State Primary Healthcare Development Agency (ASPHCDA) introduced ALMANACH (Algorithms for the MANAgement of Childhood Illness) in Adamawa State, Nigeria [23]. Adamawa, one of Nigeria’s poorest state with a poverty rate of 74% [24], has been deeply affected by the Boko Haram insurgency. In the aftermath of this crisis, during which numerous healthcare workers (HW) either fled or were killed, ALMANACH, a Clinical Decision Support System (CDSS) designed for use on tablet [25], was perceived as a strategy to support low-level health staff. A CDSS is a digital tool that helps healthcare providers make evidence-based clinical decisions by offering guidance, alerts, or recommendations during patient care [26,27].

ALMANACH, based on the Integrated Management of Childhood Illness (IMCI) protocols [28], incorporates updated diagnostic algorithms, new diagnostic tools, and is tailored to the local epidemiological profile and HWs. By guiding health providers step-by-step through the consultation, ALMANACH enables immediate data entry and real-time monitoring, as all actions—symptoms, findings, and clinical decisions—are recorded and uploaded throughout the encounter. This feature benefits HWs by reducing the time required for documentation and allows health managers to monitor service delivery. Data from ALMANACH are integrated with the District Health Information System 2 (DHIS 2) [29], the same platform used to manage data collected by Adamawa’s RHIS. However, ALMANACH automatically uploads data whenever an internet connection is available, whereas RHIS depends on manual data entry from paper clinic registers, which is carried out monthly at the beginning of the subsequent month to report on the previous period. This manual entry is often perceived as a burdensome task and carried on by undertrained staff, leading to frequent typographical errors [30,31]. A more detailed description of ALMANACH has been provided elsewhere [25,32].

**Fig 1:**
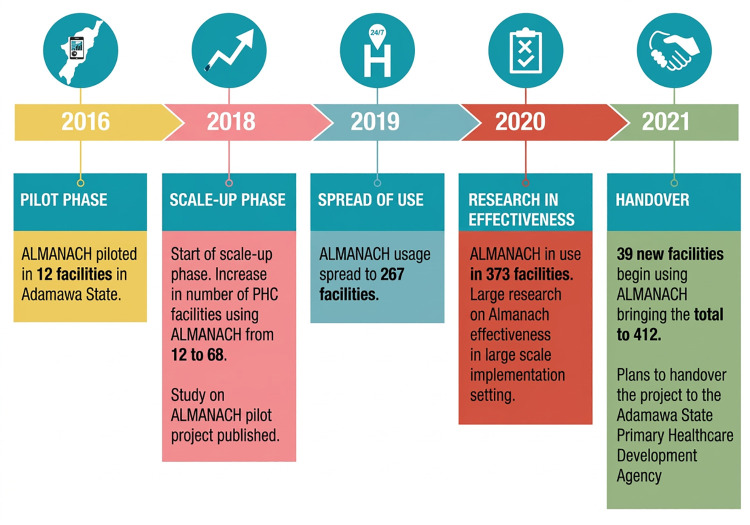
Phased timeline for bringing ALMANACH to scale in Adamawa State [35].

An initial evaluation of ALMANACH in Adamawa found that the tool improved the quality of paediatric care while simultaneously reducing antibiotic prescriptions [33]. Moreover, an observational study conducted in 2020 showed higher recovery rates among children treated using ALMANACH [34]. ALMANACH scaling up started in 2018 with the goal to cover all 412 Primary Health Care Centers (PHCC) present in Adamawa State (Fig 1). Although ALMANACH had been previously implemented in Afghanistan [36] and is currently in use in Somalia [37] and Libya [38], this represented its most extensive deployment to date.

Regarding the implementation context, Adamawa provides 24/7 primary care, with PHCCs generally similar in layout, equipment, and staffing. Most have a consultation room, delivery room, small ward (~6 beds), drug store, and bathroom, often with labs, family planning rooms, and dispensaries. Consultations are mainly done by Community and Junior Community Health Extension Workers, though supervision by a medical officer—required by job descriptions—is usually lacking [39]. Nigeria’s skilled HW density is only 1.95 per 1,000, far below international standards [40]. Before ALMANACH, a survey found poor conditions: consultations lasted 1–2 minutes with no triage, antibiotics were overprescribed (77.7% of children; 80.3% without clear justification), preventive care was inconsistent, fewer than half of children received proper examinations (45.5%), and 70.5% of treatments were inappropriate [28].

Evidence shows that ICT can enhance RHIS performance [41,42], including in LMICs [29,43,44]. Furthermore, a recent systematic review has documented the benefits of digital adaptations of IMCI in improving the quality of care [45]. However, there is scarce evidence on how these digital health interventions interact with RHIS. This gap persists despite repeated calls to strengthen interoperability—that is, the ability of distinct digital platforms, software, and databases to exchange, interpret, and use data seamlessly, without manual re-entry or loss of information integrity [46–49].

This study aimed to assess the concordance and divergence between ALMANACH and the Adamawa RHIS for malaria, pneumonia, gastrointestinal disorders (mainly acute watery diarrhoea), and measles in children under five years of age. If consistency between the two systems were confirmed, ALMANACH could help improve the timeliness and integration of clinical data flows while reducing duplication of reporting tasks. These potential benefits, however, should not be interpreted as evidence of superior data quality, as no independent assessment of the accuracy of either system was available.

## Methods

### Ethics statement

This study used de-identified, aggregated programme data collected for service evaluation in partnership with the Adamawa State Primary Health Care Development Agency (ASPHCDA). No individual-level identifiers were analysed. Ethical approval for the study was obtained from the Health Research Ethics Committee of Adamawa State (reference S/MOH/1131/I) and the Kantonale Ethikkommission Bern (reference Req-2025-00728).

### Study design and setting

We conducted a retrospective, facility-level comparative analysis between data collected from ALMANACH and RHIS covering consecutive quarterly periods from January 2017 through December 2021. During this period, ALMANACH recorded 250,324 clinical consultations for children aged 2–59 months. The project was scaled up from the initial 12 pilot PHCCs to all 398 PHCCs in Adamawa, as well as 15 outpatient departments in general hospitals. Fourteen PHCCs were excluded from implementation due to lack of internet connectivity.

### Data sources and study population

The analytic population included facility-quarters for which both ALMANACH and the RHIS reported service counts for children under five years of age. Since ALMANACH targets children aged 2–59 months while RHIS includes all ages, only a subset of the information collected overlaps between the two systems.

Quarterly case counts for malaria, pneumonia, gastrointestinal disorders, and measles were extracted from ALMANACH and RHIS. To ensure maximum comparability, analyses were restricted to matched facility-quarters present in both datasets.

### Data processing and quality checks

Case definitions followed program standards, and sensitivity analyses examined the influence of potential definitional differences for pneumonia and measles. We screened the data for outliers, and month-to-quarter aggregation errors; suspected data-entry issues were flagged and examined, and missing values were retained as missing rather than imputed as zero. We chose to remain as close as possible to the realities of the local context and, after carefully investigating potential data-entry errors, we decided to retain and report the data exactly as they were originally collected.

### Outcome definition

We chose the facility-aggregated quarterly absolute relative difference (ARD) as the primary measure of discrepancy between the values reported by RHIS and ALMANACH [50,51]. The ARD measures the absolute difference between two values relative to their mean, expressed as a percentage. Using the average as the denominator ensures that the ARD is symmetric and mathematically bounded at a maximum of 200% (this occurs when one value is exactly double the other, as the numerator—the absolute difference—equals the denominator—the average). Ninety-five percent confidence intervals (95% CI) for the ARD were derived using non-parametric bootstrapping. Hence, the formula to calculate ARD is as follows:


$$\begin{array}{c} ARD=|(A-B)/((A+B)/2)|\times 100 \end{array}$$

As no gold standard was available, the ARD with the mean of the two systems as the reference point, provides neutral and balanced basis for comparison. We categorised ARD values as ≤60% (acceptable), 61–120% (moderate), 121–199% (high), and ≥200% (extreme). This classification was applied across the implementation period from January 2017 to December 2021 to assess to what extent discrepancies between the two systems changed over time. Graphically, for each disease, results are presented in three panels: the overall trend in case counts, the time series of ARD between the two data systems, and the distribution of PHCCs across ARD categories.

### Statistical analysis

Wilcoxon signed-rank tests (two-sided, α = 0.05) were applied at the paired facility-quarter level to compare values reported by the two systems. A statistically non-significant result (p > 0.05) indicates that no systematic difference in median counts was detected across paired observations, despite it does not necessarily imply that the two systems necessarily capture the same underlying population.

To account for longitudinal clustering, we analysed quarterly case counts for malaria, gastrointestinal disorders, pneumonia, and measles using linear mixed-effects models. The outcome was the number of cases reported per health facility per quarter. Each model included reporting system (RHIS and ALMANACH, with RHIS as the reference) and calendar quarter as fixed effects, and a random intercept for health facility to account for repeated observations within facilities. Models were fitted with restricted maximum likelihood (REML) using the lme4 [52] and lmerTest [53] packages in R (version 4.4.1) [54]. To obtain inference robust to heteroscedasticity and within-facility correlation, we calculated cluster-robust (CR2) standard errors and Satterthwaite degrees of freedom using the clubSandwich package [55]. For each fixed effect we report the estimated coefficient (difference in number of cases) with 95% Confidence Interval (95% CI), and the corresponding two-sided p-value. Since numerous quarterly comparisons were performed across the four diseases, p-values for the time effects were further adjusted using a Holm–Bonferroni correction applied globally across all diseases and quarters to control the overall family-wise error rate. Two-sided p-values <0.05 were considered statistically significant.

## Results

Reported case numbers for malaria, pneumonia, gastrointestinal disorders, and measles corresponded to facility-aggregated quarterly ARDs of 80.6% (95% CI: 64.4–89.6), 32.6% (95% CI: 17.1–47.4), 34.0% (95% CI: 32.3–48.0), and 86.2% (95% CI: 45.6–162.8), respectively. Table 1 summarized these results.

**Table 1:** Total cases reported by ALMANACH and Adamawa RHIS for malaria, pneumonia, gastrointestinal disorders and measles and their median difference and ARD (2017-2021).

	ALMANACH (#)	RHIS (#)	Median difference (95% CI)	Quarterly ARD (%) (95% CI)
Malaria	116,018	233,548	6,055 (2,401–8,627)*	80.6 (64.4–89.6)
Gatrointestinal disorders	43,003	62,412	956 (411–1,382)*	32.6 (17.1–47.4)
Pneumonia	20,980	29,416	358 (141–597)**	34 (32.3–48.0)
Measles (2017–2021)	4,355	2,508	77 (38–134)*	86.2 (45.6–162.8)
Measles (2018–2021)	3,562	2,429	69 (15.5–111.5) *	85.6 (42.3–108.8)

* p value <0.01 (Wilcoxon signed-rank test).

** p value <0.05 (Wilcoxon signed-rank test).

### Malaria

**Fig 2:**
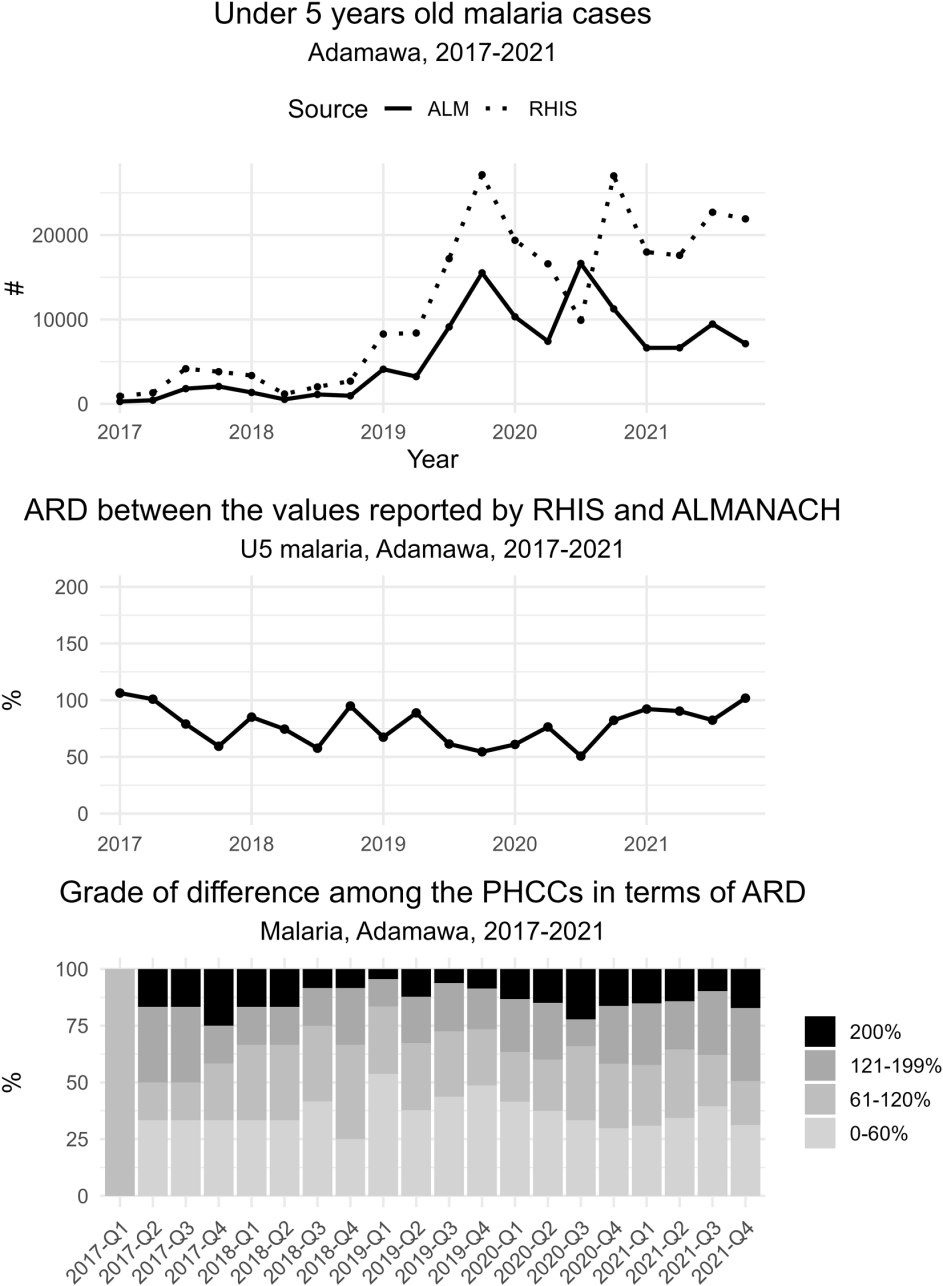
Summary of the analysis for the malaria cases notified in Adamawa (2017-2021).

Throughout the observation period, RHIS consistently reported more malaria cases than ALMANACH, with a total of 233,548 cases compared with 116,018 in ALMANACH and a median difference of 6,055 cases (95% CI: 2,401–8,627; p < 0.01). The ARD was 80.6% (95% CI: 64.4–89.6), indicating a substantial discrepancy between the two reporting systems (Table 1), although both displayed similar overall trends. RHIS showed a steeper increase, peaking at the end of 2020, whereas ALMANACH reached its peak earlier, in mid-2020 (Overall trend in case counts, (top line graph), Fig 2). The ARD fluctuated consistently between 50% and 100% (Time series of ARD (middle line graph), Fig 2). When analysing individual PHCCs, the percentage of facilities with an ARD above 120% began to increase after 2019 showing a high discrepancy between the two data collection systems. This upward trend persisted throughout the implementation period (Distribution of PHCCs across ARD categories (bottom stacked bar chart), Fig 2). The linear mixed-effects model estimated that RHIS reported on average 40.2 (95% CI: 31.1–49.3, p < 0.01) more cases per health facility and quarter than ALMANACH. Using cluster-robust standard errors, the difference remained highly significant indicating a consistent and substantial reporting gap between the two systems.

### Gastrointestinal disorders

**Fig 3:**
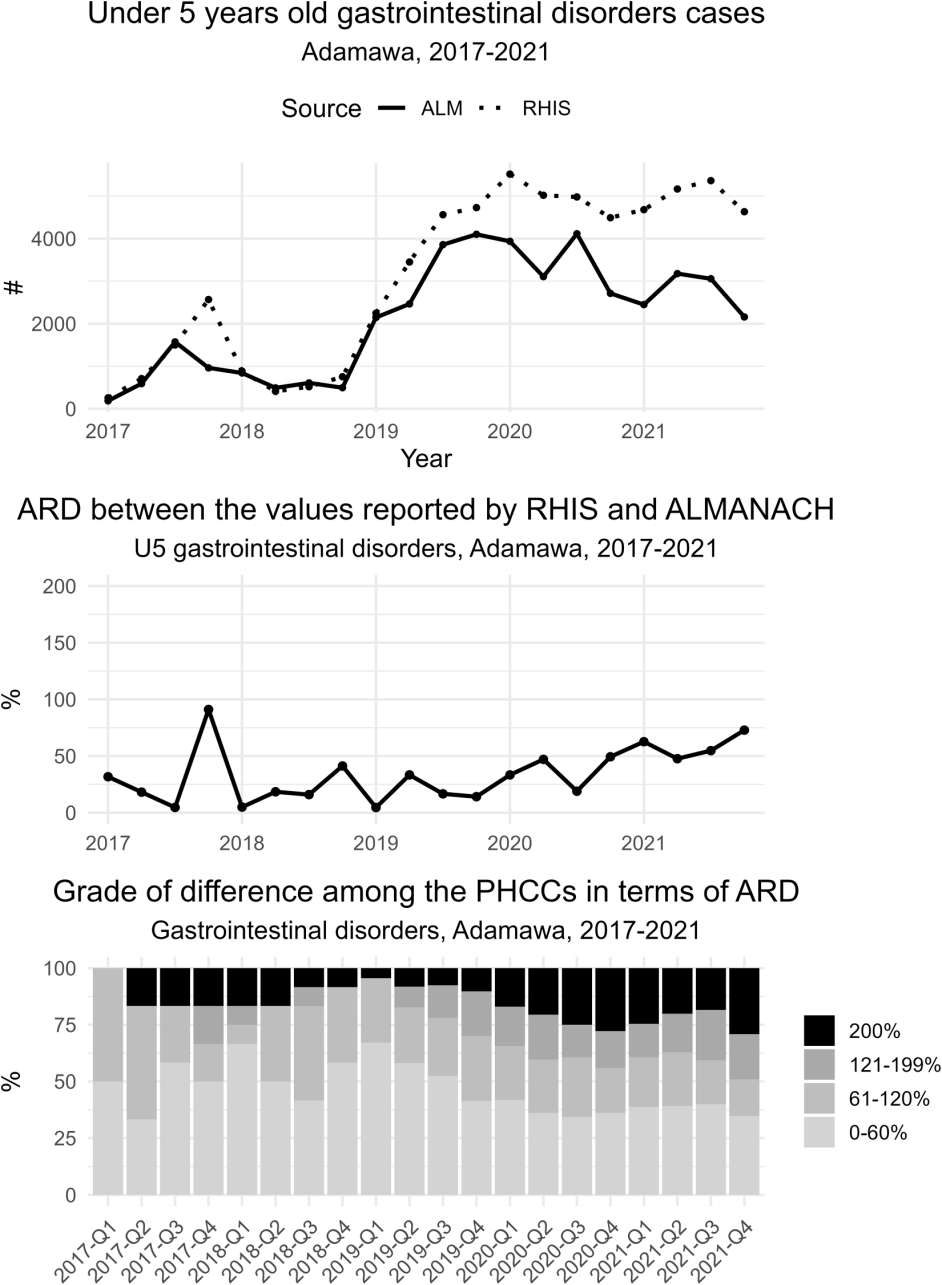
Summary of the analysis for the gastrointestinal disorder cases notified in Adamawa (2017-2021).

RHIS reported more gastrointestinal disorders (62,412 vs 43,003) with a median difference of 956 cases (95% CI: 411–1,382; p < 0.01). The ARD was 32.6% (95% CI: 17.1–47.4) (Table 1). Both systems showed a steady increase in reported cases from 2017 to 2020, peaking in 2020, with particularly strong concordance between systems during 2018–2019. Post-2020, case numbers appeared to stabilize or slightly decrease. Notably, RHIS consistently reported higher gastrointestinal disorders than ALMANACH throughout the period (Overall trend in case counts (top line graph), Fig 3).

The ARD between RHIS and ALMANACH fluctuated over the years, with pronounced peaks in certain periods, particularly at the end of 2017, where discrepancies between the systems were at their highest. Although ARD values varied, they seldom reached zero, indicating persistent discrepancies in reporting (Time series of ARD (middle line graph), Fig 3). However, ARD values rarely exceed 100%. From 2019 to 2021, ARD values were lower, suggesting improved alignment between the two data sources.

At the PHCC level, ARD values below 120% were predominant; however, the proportion of PHCCs with 200% discrepancies began to rise starting in 2020 (Distribution of PHCCs across ARD categories (bottom stacked bar chart), Fig 3). The linear mixed-effects model showed that RHIS reported on average 6.6 (95% CI: of 4.3–8.0, p < 0.01) more cases per health facility and quarter than ALMANACH.

### Pneumonia

**Fig 4:**
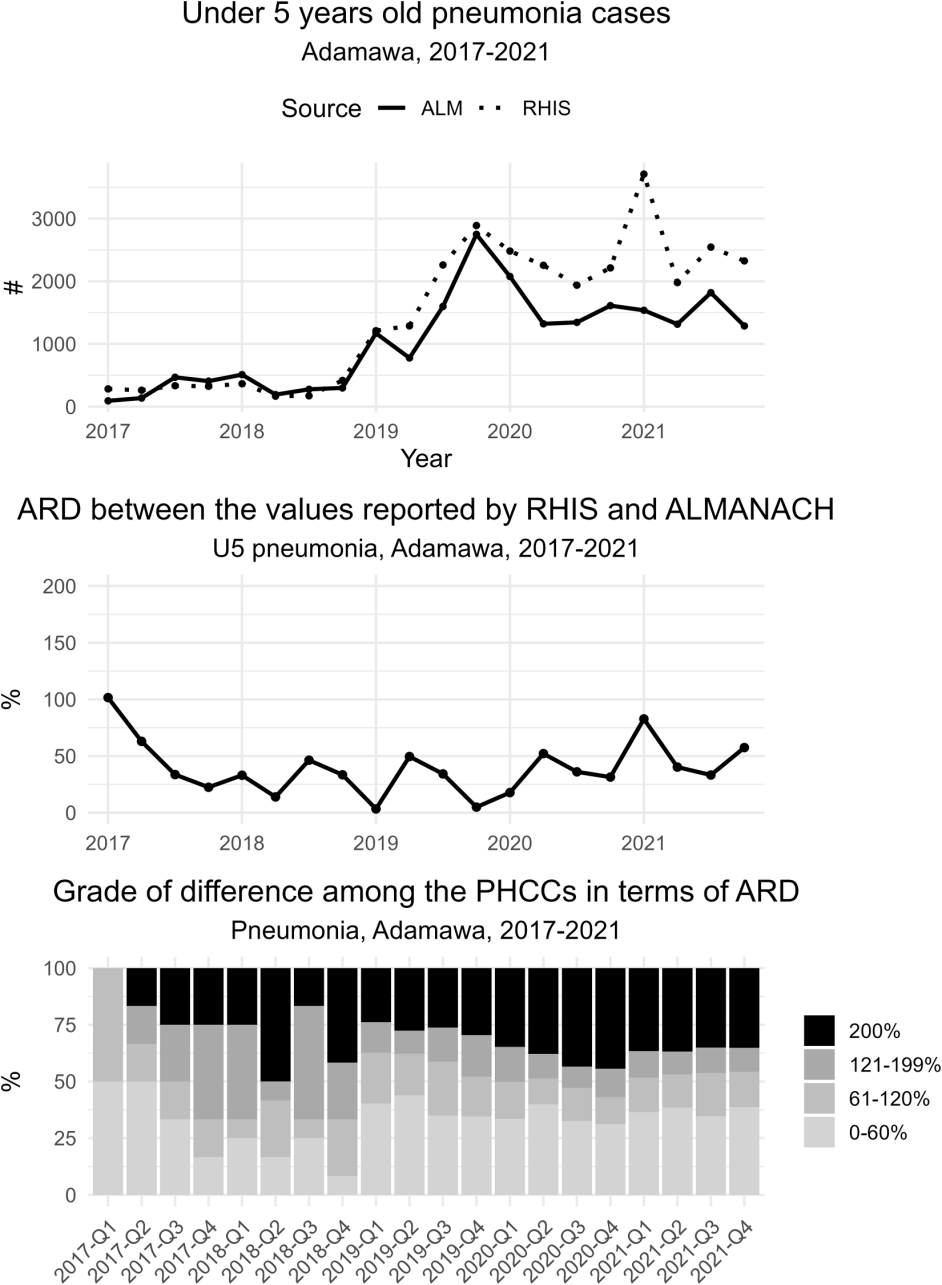
Summary of the analysis for the pneumonia cases notified in Adamawa (2017-2021).

RHIS reported 29,416 cases compared with 20,980 cases recorded by ALMANACH. The median difference between the two systems was 358 cases (95% CI: 141–597; p < 0.05), indicating consistently higher counts in RHIS. The ARD was 34.0% (95% CI: 32.3–48.0), demonstrating a substantial but slightly smaller discrepancy than that observed for malaria and gastrointestinal disorders (Table 1). Up to the end of 2019 both ALMANACH and RHIS reported a similar number of pneumonia cases. From 2020 onward, RHIS consistently recorded higher counts than ALMANACH, including a peak in 2021 that was not captured by ALMANACH (Overall trend in case counts (top line graph), Fig 4).

The ARD began at a high level (around 100%) in 2017 but gradually decreased at acceptable level, reaching a lower and more stable level by 2019, with slight fluctuations thereafter. In 2021, a minor upward trend in ARD suggests a slight increase in divergence between the systems (Time series of ARD (middle line graph), Fig 4).

At the PHCC level, discrepancy levels varied over time. Certain quarters, such as mid-2018 and early 2019, showed a wider distribution of cases across moderate and high discrepancy levels, while others were dominated by the highest discrepancy category (200%). In quarters like 2017-Q2 and 2021-Q2, there is a relatively larger proportion in the lower discrepancy categories (0–60% and 61–120%), indicating periods of improved alignment between the systems (Distribution of PHCCs across ARD categories (bottom stacked bar chart), Fig 4). For pneumonia, RHIS reported on average 2.9 more cases per health facility and quarter than ALMANACH (95% CI: 1.2–4.6, p < 0.01).

### Measles

**Fig 5:**
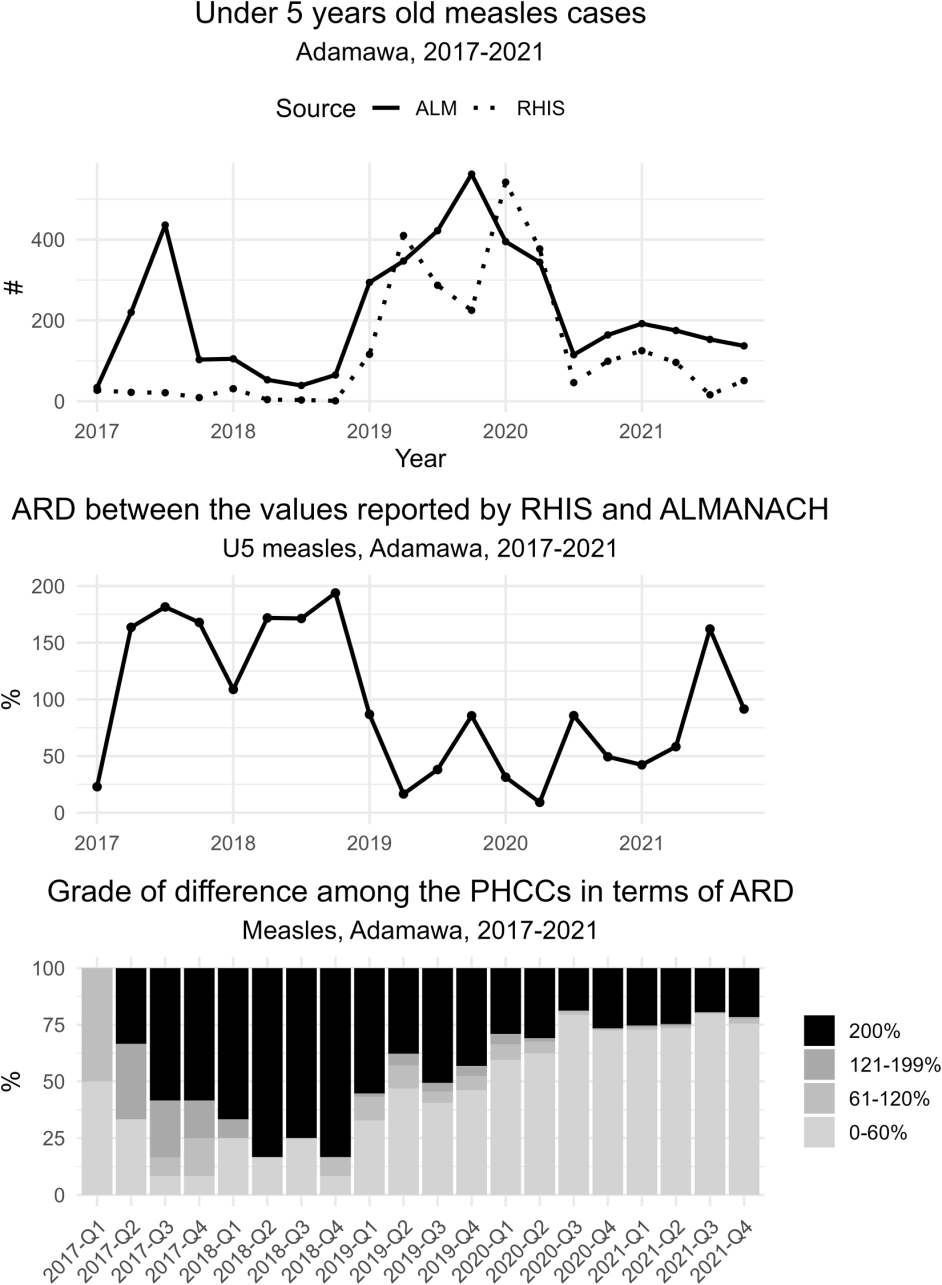
Summary of the analysis for the measles cases notified in Adamawa (2017-2021).

RHIS reported 2,508 cases compared with 4,355 cases recorded by ALMANACH. The median difference was 77 cases (95% CI: 38–134; p < 0.05), indicating that ALMANACH consistently reported more cases than RHIS. The ARD was 86.2% (95% CI: 45.6–162.8). ALMANACH data shows a sharp peak in measles cases in 2017, surpassing 400 cases. This spike is not reflected in RHIS data, which remained comparatively low and stable. From 2018 onward, both systems indicate an upward trend in measles cases, with a notable peak around 2020. Although RHIS showed a slightly delayed increase compared to ALMANACH, by 2020, both systems reported relatively high case counts, suggesting alignment in trend capture during this period. Following the 2020 peak, both ALMANACH and RHIS reported a decline, with case numbers stabilizing at a lower level by 2021. Despite these trends, ALMANACH consistently reported higher measles case counts than RHIS throughout the observed period (Overall trend in case counts (top line graph), Fig 5).

The ARD began relatively low in 2017 but rose sharply to nearly 200%, indicating substantial discrepancies between RHIS and ALMANACH. From 2017 to 2019, the ARD remained high, fluctuating around 150–200%, before dropping to acceptable level around 50%. After 2019, the ARD continued to fluctuate with some increases and decreases but remained lower on average compared to earlier years (Distribution of PHCCs across ARD categories (bottom stacked bar chart), Fig 5).

At the PHCC level, an ARD of 200% was consistently present across most quarters, with some periods showing more extreme discrepancies (e.g., 2017-Q3, 2018-Q2) and others less. After 2020, these extreme discrepancies gradually decreased (Distribution of PHCCs across ARD categories (bottom stacked bar chart), Fig 5). For measles, RHIS reported on average 0.6 fewer cases per health facility and quarter than ALMANACH (95% CI: –1.2 to –0.07, p = 0.029).

Recalculating the model after excluding 2017, the year affected by a falsely reported epidemic, showed no substantial change: RHIS reported on average 0.4 fewer cases per health facility and quarter than ALMANACH (95% CI –1.2 to –0.07, p < 0.01).

Table 2 summarizes the effect estimates for RHIS vs ALMANACH for each disease, with robust standard errors (CR2), 95% CI, and Holm-adjusted p-values. All diseases except measles show a statistically significant positive difference, indicating RHIS reports more cases than ALMANACH; for measles the effect is slightly negative but also statistically significant.

**Table 2:** Summary of RHIS vs ALMANACH Effects. Linear mixed-effects models were fitted separately for each disease. The table reports the estimated difference in number of cases per health facility and quarter (RHIS minus ALMANACH) with 95% confidence intervals, cluster-robust (CR2) standard errors, and Holm-adjusted p-values. All p-values are based on Satterthwaite degrees of freedom (~276).

Disease	Estimate (95% CI)	Robust Standard Errors	Holm-adjusted p
Malaria	40.2(31.1–49.3)	4.58	<0.01
Gastrointestinal disorders	6.6 (4.3–8.0)	1.17	<0.01
Pneumonia	2.9 (1.2–4.6)	0.88	<0.01
Measles (2017–2021)	−0.63 (−1.2– − 0.07)	0.29	0.029

## Discussion

The primary objective of ALMANACH was to enhance the quality of paediatric care and to demonstrate its effectiveness in real-world settings—an aim that was successfully achieved in Adamawa [33,34]. However, our findings reveal a systematic divergence between RHIS and ALMANACH data that varies by disease and over time, and that widens as ALMANACH continues to scale up.

ALMANACH played a pivotal role in driving change to boost quality of care at primary health care [36], and its use has been demonstrated to be safe [34]. To support its implementation, supervision was incorporated into the responsibilities of the existing Integrated Supportive Supervision team promoted by the World Bank. These supervision activities included monthly data verification and monitoring visits to track changes in practice after implementation, regular supervision meetings, quarterly reporting, and action-oriented decision-making [56]. A dedicated Project Implementation Unit was established within the ASPHCDA, supported by a specific budget line in the agency’s annual operational plan, to sustain project outcomes and ensure long-term sustainability [57]. During first year of implementation, data were routinely and closely monitored to assess adoption of the tool, and regular discussions were held with key stakeholders to identify challenges and potential bottlenecks. Furthermore, no major changes occurred in the Adamawa population or in the data-collection process during the implementation period. However, our current analysis highlights aspects of our approach that could have benefited from further refinement.

A preliminary assessment of the RHIS in Adamawa was not conducted prior to the implementation of the digital intervention, partly because reinforcing the local RHIS was not initially planned, as ALMANACH was viewed as a feasible alternative. Although both systems upload their data to DHIS2, they operate independently, and their datasets are not consolidated into a single account. As a result, data flows remain unintegrated and require separate analysis at the health management level.

This lack of integration has resulted in a misalignment between many ALMANACH indicators and those institutionally reported by the RHIS. In addition, the RHIS covers all children under five years of age and includes diagnoses not captured by ALMANACH (e.g., fractures, wounds, burns), whereas ALMANACH focuses on children aged 2–59 months and addresses only IMCI-related conditions. Moreover, while ALMANACH digitizes data at the point of care, the national RHIS remains paper-based up to the Local Government Area level, which limits local health managers’ access to timely RHIS data and further hinders integration between the two systems.

Only four indicators, malaria, gastrointestinal disorders, pneumonia, and measles, were captured by both systems. The way ALMANACH captures pneumonia and measles diagnoses may have contributed to some of the observed discrepancies. Unlike standard clinical visits without digital support, ALMANACH distinguishes pneumonia from other respiratory infections by analysing breathing patterns and supports measles diagnosis through a built-in dermatological atlas. In 2017, however, the tool was initially incorrectly used, leading to a falsely reported measles outbreak. Several HWs entered consultation data at the end of the day rather than during each patient visit, paying insufficient attention to case details. The implementation team subsequently reinforced the requirement to use ALMANACH in real time during consultations, and no similar incidents have occurred since. Nevertheless, the continued use of ALMANACH has been accompanied by persistently high numbers of measles diagnoses, raising the possibility that the built-in dermatological atlas may encourage measles reporting and potentially increase false positives. On another hand, given the documented habit of antibiotics over-prescription, pneumonia may have been over diagnosed in routine practice [36], making ALMANACH case count potentially closer to true incidence, although this cannot be confirmed without an external quality comparator.

Other factors may also help explain the discrepancies between the two systems, particularly the consistently lower number of malarial, gastrointestinal disorder, and pneumonia cases reported by ALMANACH compared with RHIS. Trained staff often failed to share their knowledge with colleagues, limiting the adoption of the tool. High staff turnover further exacerbated the issue. Although the tablets were equipped with educational materials and a WhatsApp group was created to facilitate communication, these challenges persisted. Additional barriers to the adoption of the digital tool included longer consultation times, misconceptions about appropriate prescribing practices, and the scarce involvement of HWs in the development of the algorithms — a factor that undermined their confidence in the digital intervention. Finally, ALMANACH was mandatory, but it was lacking enforcement, and supervision became increasingly difficult as more health facilities were included over the course of the implementation period. Ideally, an 80% utilization rate was hypothesized as the target for ALMANACH. However, no PHCC was able to achieve this target.

The RHIS in Adamawa was neither accurate nor reliable. One-third of the PHCCs, severely affected by the Boko Haram insurgency, experienced reporting gaps and the influx of internally displaced people left the population size in Adamawa State unknown, and, due to a technical glitch, it was not possible to extract the total number of under-five patients accessing curative care. Additionally, the RHIS faced challenges typical of many systems in LMICs: frequent delays in reporting, missing monthly reports, and a lack of mechanisms to ensure data consistency [46]. Moreover, we cannot rule out the possibility that health staff viewed the increased digitization as enhanced oversight of their work, potentially leading to resistance to using the tool.

Based on our experience, several strategies can enhance the implementation and adoption of digital tools like ALMANACH. To foster engagement, behavioral strategies such as gamification can be employed, while recognizing and rewarding active users—through certificates, awards, or public acknowledgment—can reinforce motivation [58]. To improve oversight and support, developers could include features to track individual user activity, enabling targeted supervision and tailored training. In our project, however, a shared login was used within each facility, limiting the ability to monitor usage patterns at the individual level. Linking the use of CDSS to professional development—such as integrating ALMANACH training into health-worker curricula—or to performance-based incentives may enhance motivation and uptake. Identifying early adopters and empowering them as peer mentors or champions can further promote adoption across teams. In September 2019, the ALMANACH concept and training were incorporated into the training curriculum for Community Health Extension Workers and midwives [57]. During this training process, it is crucial to present digital tools as supportive aids rather than a substitution for clinical judgment, thereby reinforcing the central role of HWs in decision-making. Complementary training in epidemiology and data use—whether before, during, or after implementation—can further build confidence and competence.

The mechanisms we observed—such as inconsistent adoption of ALMANACH, staff turnover, limited supervision, perceptions of increased oversight, and workflow constraints—are consistent with patterns widely reported in the implementation of digital CDSS across LMICs. Several studies have shown that digital IMCI tools improve adherence to clinical guidelines [59], reduce inappropriate antibiotic use [60], and enhance the overall quality of paediatric assessment [45]. However, these tools also face barriers including additional workload generated by parallel reporting systems [61,62], connectivity limitations [63,64], gaps in supportive supervision [65], and concerns among HWs about increased monitoring and accountability [62]. Similarly, the broader literature on RHIS strengthening highlights persistent weaknesses in the completeness, timeliness, and accuracy of paper-based routine data, which often coexist with digital systems and contribute to inconsistent reporting and limited data use [3,13,66,67]. Situating our results within this wider evidence base suggests that the discrepancies between ALMANACH and the RHIS observed in Adamawa are not unique, but reflect structural challenges common to ICT interventions in LMICs [68]. Rather than assuming that alignment of the two systems would resolve these inconsistencies, our findings point to a more fundamental challenge: the need for reliable, high-quality routine data to support decision-making. Both ALMANACH and the RHIS represent potential sources of valuable information, but neither currently provides a fully accurate representation of disease trends. Addressing this gap will require targeted investments in data-quality verification, workflow optimisation, and strengthened data-use practices within facilities and across the health system.

Our study presents several important limitations. First, there is no gold standard against which to judge data quality; we therefore quantify divergence rather than infer superiority. Second, differences in case definitions and age bands (ALMANACH 2–59 months vs RHIS under-five) may create structural discordance, particularly for pneumonia and measles. Third, residual misclassification, delays in RHIS reporting, and inconsistent ALMANACH use during scale-up may introduce bias in the comparisons. Fourth, although paired analyses and mixed-effects models were applied, unmeasured facility-level factors such as workload, staffing, and supervision could still confound differences. Finally, some sensitivity analyses relied on aggregated denominators that were unavailable.

## Conclusion

The findings of this study reflect the structural weaknesses that hinder the availability of quality health data in many LMIC settings.

In Adamawa, ALMANACH was the first project to introduce digital data collection at the point of care. While its digital approach may be better suited to a context with existing high levels of digitization, its benefits remain noteworthy. It is unlikely that digitization alone could significantly enhance system performance if the underlying manual or paper-based system is already underperforming. However, since the handover of ALMANACH to the ASPHCDA, the state-level antibiotic prescription rate has reportedly decreased from 78% in 2018–19–21% in 2023 [57], demonstrating its positive impact on prescribing practices and quality of care.

While ICT solutions offer great potential for improving healthcare in LMICs, as evidenced by various studies [45,69,70], careful joint planning from the outset is essential to ensure full integration with local health systems and the sustainability of these interventions. Key considerations should include integrating and utilizing data collection systems, allocating specially trained staff, reorganizing workloads, and ensuring adequate funding and resource allocation [71,72].

A stronger culture of data use should accompany future ICT initiatives. Empowering health staff to collect data while reducing the burden of manual reporting can further encourage acceptance of new technologies and promote their sustained use. Future field research should incorporate qualitative and clinical verification methods—such as direct observation, chart review, or interviews—to better understand the sources of discrepancies between digital data collection and RHIS.
